# Exosome‐derived uterine miR‐218 isolated from cows with endometritis regulates the release of cytokines and chemokines

**DOI:** 10.1111/1751-7915.13565

**Published:** 2020-03-30

**Authors:** Xiangguo Wang, Xinxin Yao, Tongtong Xie, Zhenyu Chang, Yong Guo, Hemin Ni

**Affiliations:** ^1^ Animal Science and Technology College Beijing University of Agriculture Beijing 102206 China

## Abstract

As an inflammation of the endometrium, endometritis can affect fertility and lead to serious economic losses in the dairy industry. Widely found in various tissues and body fluids, exosomes and exosome micro (mi)RNAs have been shown to play an important regulatory role in the immune responses. As one of differentially expressed exosome miRNAs, miR‐218 is involved in the pathogenesis of bovine endometritis. The mechanisms of miR‐218 in regulating the release of cytokines and chemokines in endometritis, however, are poorly understood. Exosomes were isolated from bovine uterine cavity fluid and verified by transmission electron microscopy. An *in vitro* lipopolysaccharide‐treated cell model for bovine endometritis was then established to evaluate the correlation between exosome‐derived miR‐218 and the immune responses. We demonstrated that exosomes could be used to deliver miR‐218 from endometrial epithelial cells (EECs) into the uterine microenvironment and adjacent recipient cells to modulate local immune responses. miR‐218 packaged in the exosomes secreted from EECs acts as an inhibitor by blocking immune factors such as interleukin (IL)‐6, IL‐1β, tumour necrosis factor‐α, the chemokines macrophage inflammatory genes *(MIP)‐1α* and *MIP‐1β* to maintain the immune balance in the uterus. However, uterine inflammation altered the immunoregulatory mechanism of exosome miR‐218. MiR‐218 is a potential biomarker for the detection of endometritis. Our findings also revealed a new mechanism for the development of endometritis in cows.

## Graphical abstract

Exosomes could be used to deliver miR‐218 from endometrial epithelial cells (EECs) into uterine microenvironment and adjacent recipient cells to modulate local immune responses. MiR‐218 packaged in exosomes secreted from EECs acts as an inflammatory inhibitor by blocking immune factors and chemokines. MiR‐218 is a potential biomarker for monitoring the pathogenesis of bovine endometritis.

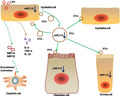

## Introduction

As an inflammation of the endometrium, bovine endometritis is associated with lower conception rates, increased intervals from calving to first service, and more culls for failure to conceive, leading to serious economic losses in the dairy industry (Hussain and Daniel, 1991). Endometritis usually occurs after 21 days postpartum, without systemic symptoms (Bondurant, 1999). Histologically, the endometrial epithelial cells disintegrated, and the surface layer of the endometrium had different degrees of lymphocyte and plasma cell aggregation, inflammatory cell infiltration, vascular hyperaemia and stroma oedema (Bondurant, 1999). Without systemic symptoms, clinical endometritis is characterized by purulent (> 50% pus) uterine secretions which are detectable from the vagina after 21 days postpartum, or mucopurulent (about 50% pus, 50% mucus) uterine secretions from the vagina after 26 days postpartum (Sheldon, *et al.*, 2006); while subclinical endometritis is characterized by no purulent discharge from the vagina, with polymorphonuclear (PMN) > 18% in the uterine cytology samples at 21–33 days postpartum or PMN > 10% in the uterine cytology samples after 34–47 days (Kasimanickam, *et al.*, 2004).

Bovine endometrial epithelium cells (EECs) are the first line of defence to resist infection caused by various invading agents (Piras, *et al.*, 2017), depending on innate immune systems (Wira and Fahey, 2004). Signalling pathways have been well documented that regulate the response of pathogenic microorganisms to endometritis. Toll‐like receptor 4 (TLR4) can recognizes lipopolysaccharide (LPS) binding on the myeloid differentiation factor 2 on the cell surface (Kim, *et al.*, 2007; Cronin, *et al.*, 2012). TLR4–LPS binding can lead to the activation of the nuclear factor κB (NF‐κB) and mitogen‐activated protein kinase (MAPK), and the NF‐κB pathway can activate downstream inflammatory mediators including the cytokines IL‐1β and IL‐6, tumour necrosis factor (TNF)‐α, cyclooxygenase‐2, inducible nitric oxide synthase and chemokine IL‐8 (Regueiro, *et al.*, 2009; Lv, *et al.*, 2015). Moreover, MAPK pathways, including the extracellular signal‐regulated kinase 1/2, p38 and c‐Jun NH2‐terminal kinase, have been reported to regulate the LPS‐induced expression of TNF‐α (Yoon, *et al.*, 2010).

The transfer of information between cells is achieved by direct contact and cytokines. Over the past decade, however, extracellular vesicles have been recognized as the potent vehicles of intercellular communication because of their capacity for transferring proteins, lipids and nucleic acids. This can influence various physiological and pathological functions of both recipient and parent cells. As a subset of the extracellular vesicles released by almost all types of cells, exosomes contain a variety of biological components including membrane proteins, lipids, RNA and DNA (Yanez‐Mo, *et al.*, 2015). Upon being released from cells, exosomes distribute in biological fluids and are taken up by cells of the same or a different type, which then interact with exosomes and undergo biological functions (Sahoo and Losordo, 2014). Exosomes from T cells, B cells, dendritic cells (DCs) and macrophages mediate either immune stimulation or modulation (Agarwal, *et al.*, 2014). Their immunological activities affect both innate and adaptive immunity, including antigen presentation, T‐cell activation, T‐cell polarization into regulatory T cells, immune suppression and anti‐inflammatory action (Zhang, *et al.*, 2014). One study has shown that antigen presentation through exosomes plays an important role in mounting and stimulating immune responses (Natasha, *et al.*, 2014). Exosomes secreted from professional antigen‐presenting cells (APCs), that is B lymphocytes and DCs, are enriched in major histocompatibility Class‐I and ‐II complexes and costimulatory molecules, which have key functions in immunoregulation by possessing antigenic peptides (Chaput, *et al.*, 2004; Beach, *et al.*, 2014). In recent years, the effects of isolated APC‐derived exosomes have been more extensively studied for their immunoregulatory capacities *in vitro* and *in vivo* (Buschow, *et al.*, 2010).

Exosome micro (mi)RNAs, the main molecules playing a regulatory role in exosomes, primarily cause gene silencing in receptor cells (Sato, *et al.*, 2017). A mechanism of the transfer of exosome‐shuttle miRNAs among DCs was previously documented as a means of communication and post‐transcriptional regulation (Montecalvo, *et al.*, 2012). Moreover, anti‐miR‐150 molecules released by B lymphocytes were shown to be internalized by CD8^+^ T lymphocytes during cross‐priming *in vitro* and *in vivo*, resulting in the marked downregulation of endogenous miR‐150 (Almanza, *et al.*, 2013). B cell‐derived exosomes were also shown to be useful carriers to deliver anti‐miR‐155 to macrophage cell lines, thus downmodulating endogenous miRNA in recipient cells (Momen‐Heravi, *et al.*, 2014). Ismail et al. found that RNA molecules contained in macrophage‐derived exosomes were transported to target cells including monocytes, endothelial cells, epithelial cells and fibroblasts (Ismail, *et al.*, 2013), whereas Okoye et al. reported that the exosome‐mediated transfer of Let‐7d from Treg cells to Th1 cells contributes to the suppression and prevention of systemic disease (Okoye, *et al.*, 2014).

We previously found that exosome‐derived uterine miRNAs isolated from cows with endometritis impeded blastocyst development (Wang, *et al.*, 2019). The next‐generation sequencing previously showed that miR‐218 has differential expressions among the exosomes from the EEC supernatants of healthy cows and those with endometritis. However, the acting mechanism of endometrial exosome‐derived miR‐218 in the development of endometritis is still unclear. In the present study, we investigated the function of exosome miR‐218 in modulating the immune response to endometritis.

## Results

### Localization and expression of miR‐218 in uterine tissue of healthy cows and those with endometritis

**Fig. 1:**
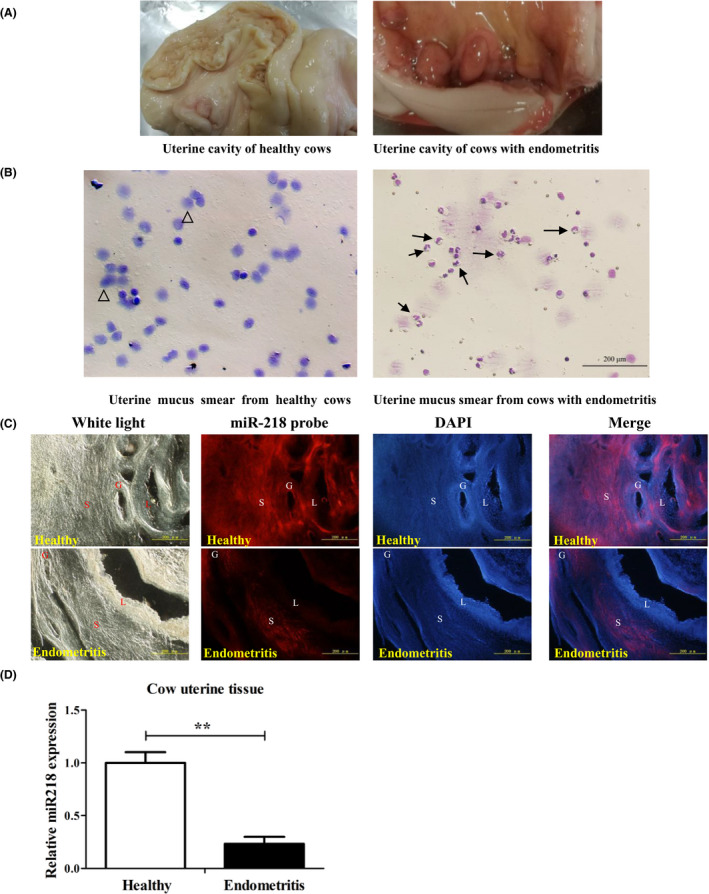
Localization and expression of miR‐218 in the uterine tissue of healthy cows and those with endometritis. A. Endometrial cavity of healthy and diseased cows. Data are representative of three independent experiments. B. Bovine uterine lavage fluid cell smear, Bar: 200 μm. Data are representative of three independent experiments. The triangle indicates the shed epithelial cells of the uterus and the arrow points for a Neutrophil. C. Localization of miR‐218 in the endometria, glands, and matrix as detected by *in situ* hybridization. S: matrix, G: gland, L: endometrial epithelium, Bar: 200 μm. Data are representative of three independent experiments. D. Expression of miR‐218 in uterine tissue of healthy cows and those with endometritis as detected with qRT‐PCR. Data are represented with mean ± standard deviation from three independent experiments, **P < 0.01.

As shown in Fig. 1A, the uterine tissue of healthy cows, which is yellowish and not inflamed, has a small amount of clear or translucent liquid flowing out of the cervix, but is free of purulent liquid. By contrast, the uterine tissue of cows with endometritis has hyperaemic oedema, red surface and reduced elasticity, with white mucopurulent secretions out of the cervix. The results showed that the cytological smears of the uterine lavage fluid from 21 to 30 days postpartum in healthy cows mainly included epithelial cells, without PMN. By contrast, a large number of PMNs appeared in the cytological smears of the uterine lavage fluid of cows with clinical endometritis, and their proportion was greater than 18% (Fig. 1B). The *in situ* hybridization revealed strong miR‐218 fluorescence localized in the uterine gland, stroma and epithelium of healthy cows. In the uterus of cows with endometritis, low miR‐218 fluorescence was only observed in the stromal tissue (Fig. 1C). The qRT‐PCR results showed that miR‐218 expression was significantly reduced in the uterine tissue of cows with endometritis compared to healthy ones (Fig. 1D, *P* < 0.01).

### Expression changes of miR‐218 in an in vitro LPS‐treated cell model for bovine endometritis

**Fig. 2:**
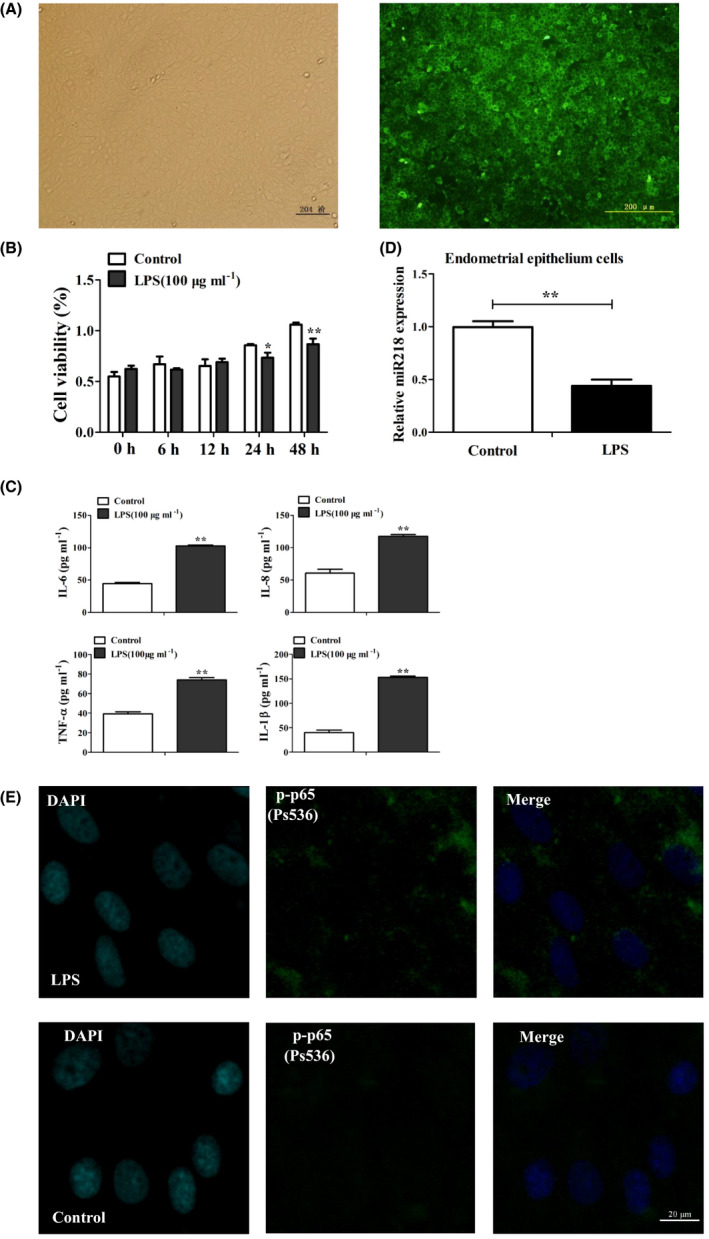
Establishment of an *in vitro* LPS‐treated cell model for bovine endometritis. A. Identification of the keratin expression in bovine endometrial epithelial cells with immunofluorescence, Bar: 200 μm. Data are representative of three independent experiments. B. Detection of the effect of LPS on endometrial epithelial cell viability with the Cell Counting Kit (CCK8). The cells were treated with 100 μg ml^−1^ of LPS for different periods of time (0–48 h) and then processed for of the analysis of cell viability. Data are represented with mean ± standard deviation from three independent experiments, *P < 0.05, **P < 0.01. C. Secretion of the immune factors IL‐6, IL‐8, TNF‐α and IL‐1β in LPS‐treated endometrial epithelial cells after 24 h as detected with ELISA. Data are represented with mean ± standard deviation from three independent experiments, *P < 0.01. D. Expression of miR‐218 in LPS‐treated endometrial epithelial cells after 24 h as detected with qRT‐PCR. Data are represented with mean ± standard deviation from three independent experiments, **P < 0.01. E. Nuclear localization of phosphorylated p65 protein in LPS‐treated endometrial epithelial cells after 24 h as detected with immunofluorescence, Bar: 20 μm.

The bovine EEC line used in this study had a uniform cell structure, showing typical epithelial cell morphology and positive keratin staining (Fig. 2A). After 24 h of 100 μg ml^−1^ LPS processing, EEC viability was significantly reduced (Fig. 2B, *P* < 0.05). Therefore, treatment with 100 μg ml^−1^ LPS for 24 h was used to simulate a cell model of bovine endometritis. In this model, the secretion of the immune factors IL‐6, IL‐8, TNF‐α and IL‐1β was significantly increased compared with the controls (Fig. 2C, *P* < 0.01). Simultaneously, the expression of miR‐218 was significantly reduced in the LPS treatment model compared with the controls (Fig. 2D, *P* < 0.01). LPS stimulation significantly promoted the phosphorylated p65 expression and nuclear localization (Fig. 2E).

### Isolation and identification of bovine uterine fluid exosomes and miR‐218 expression

**Fig. 3:**
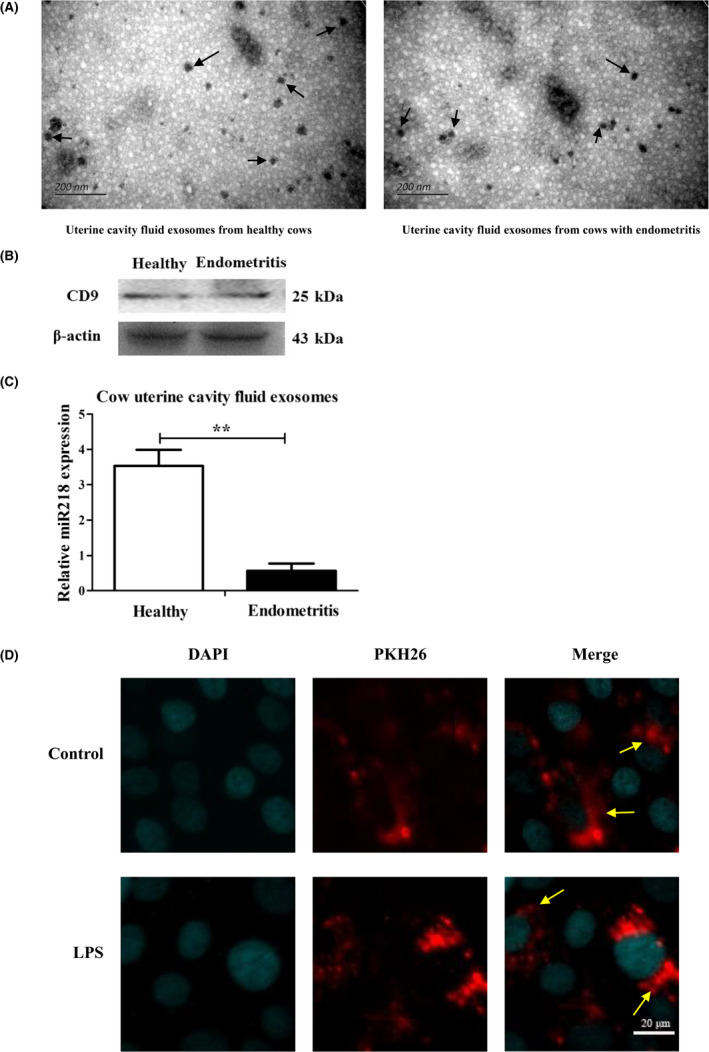
Isolation and identification of bovine uterine fluid exosomes and detection of miR‐218 expression. A. Exosomal vesicle detection with electron microscopy. Scale: 200 nm. Data are representative of three independent experiments. The arrow points for an exosome. B. Protein expression of the exosome marker CD9 as detected by western blotting. Data are representative of three independent experiments. C. Expression of miR‐218 in uterine fluid exosomes as detected with qRT‐PCR. Data are representative of three independent experiments, **P < 0.01. D. Confocal microscopy of the internalization of fluorescently labelled exosomes in EECs after 12 h incubation, Bar: 20 μm. Data are represented with mean ± standard deviation from three independent experiments. The arrow points for a PKH26 labelled exosome.

Uterine cavity exosomes extracted with the exosome extracting kit were shown by the electron microscopy to have a particle size of 30–150 nm, a cystic structure (Fig. 3A), and the positive expression of the exosome marker protein CD9 (Fig. 3B). The expression of miR‐218 in the uterine fluid‐derived exosomes of cows with endometritis was significantly reduced compared with that in exosomes from the uterine fluid of healthy cows (Fig. 3C, *P* < 0.01). Additionally, those exosomes stained by PKH26 were found to fuse into the healthy EECs and LPS‐induced inflammatory EECs when being incubated for 12 h (Fig. 3D).

### Effect of miR218 on the release of inflammatory factors in EECs

**Fig. 4:**
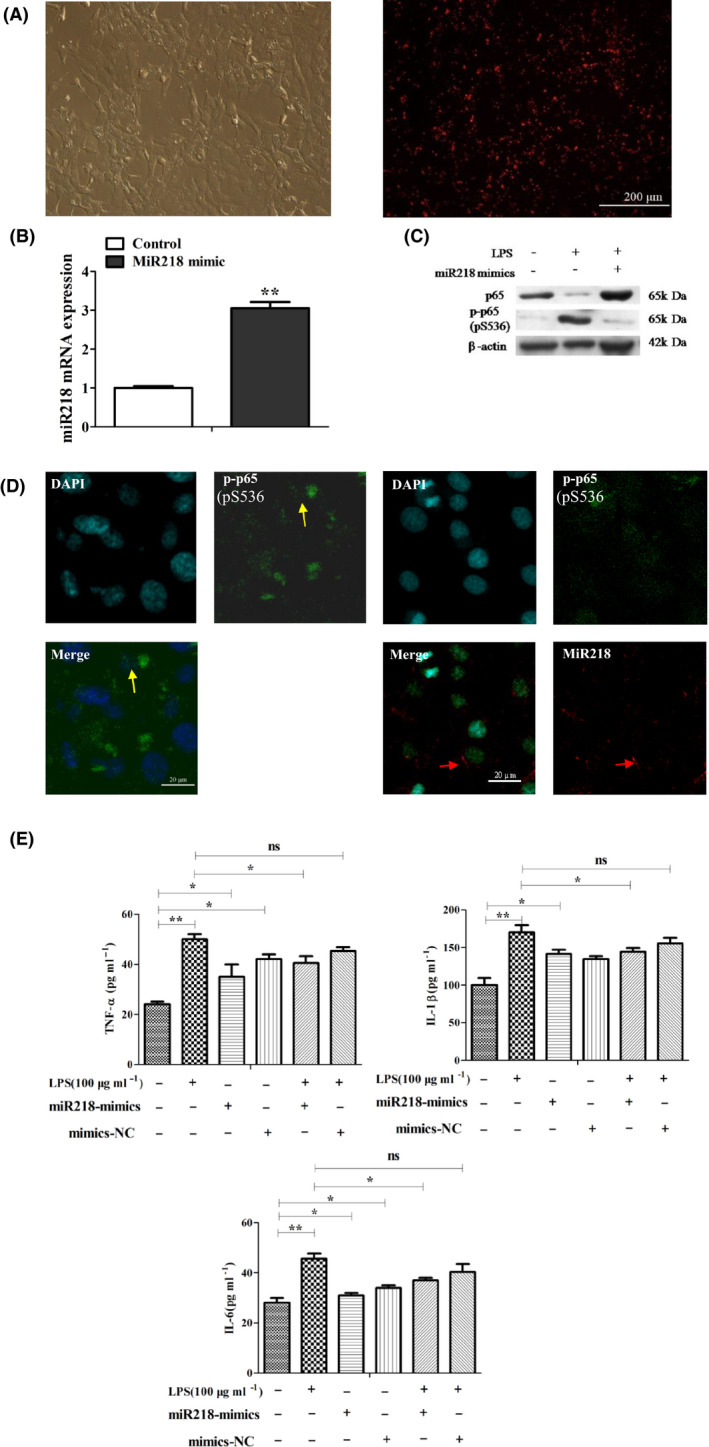
The effect of miR218 on the release of inflammatory factors in endometrial epithelial cells. A. Fluorescence imaging of miR218 mimic‐transfected EECs, Bar: 200 μm. Data are representative of three independent experiments. B. Expression of miR‐218 in EECs after the transfection of miR218 mimics for 24 h as detected with qRT‐PCR. Data are represented with mean ± standard deviation from three independent experiments, **P < 0.01. C. Western blot analysis of p65 and phosphorylated p65 in LPS‐treated EECs and miR‐218‐transfected EECs. Data are representative of three independent experiments. D. Nuclear localization of phosphorylated p65 protein in LPS‐treated endometrial epithelial cells after 24 h as detected by immunofluorescence. The yellow arrow indicates phosphorylated p65 protein (green), and the white one indicates miR‐218 (red). Bar: 20 μm. E. The effect of miR218 mimics or NC transfection on the release of inflammatory factors IL‐6, TNF‐α and IL‐1β induced by LPS administration. Data are represented with mean ± standard deviation from three independent experiments, * P < 0.05, **P < 0.01.

The transfection efficiency of the transfected miR‐218 mimics in EECs was above 98% (Fig. 4A), and the expression of miR‐218 in EECs transfected with these mimics was significantly increased (Fig. 4B, *P* < 0.01). miR‐218 mimic transfection attenuated the LPS‐induced expression of phosphorylated p65 protein (Fig. 4C), significantly reduced the LPS‐induced nuclear localization of phosphorylated p65 protein (Fig. 4D), and significantly reduced the LPS‐induced IL‐6, TNF‐α and IL‐1β secretion as well (Fig. 4E, *P* < 0.05).

### Effect of miR‐218 on the release of chemokines from EECs

**Fig. 5:**
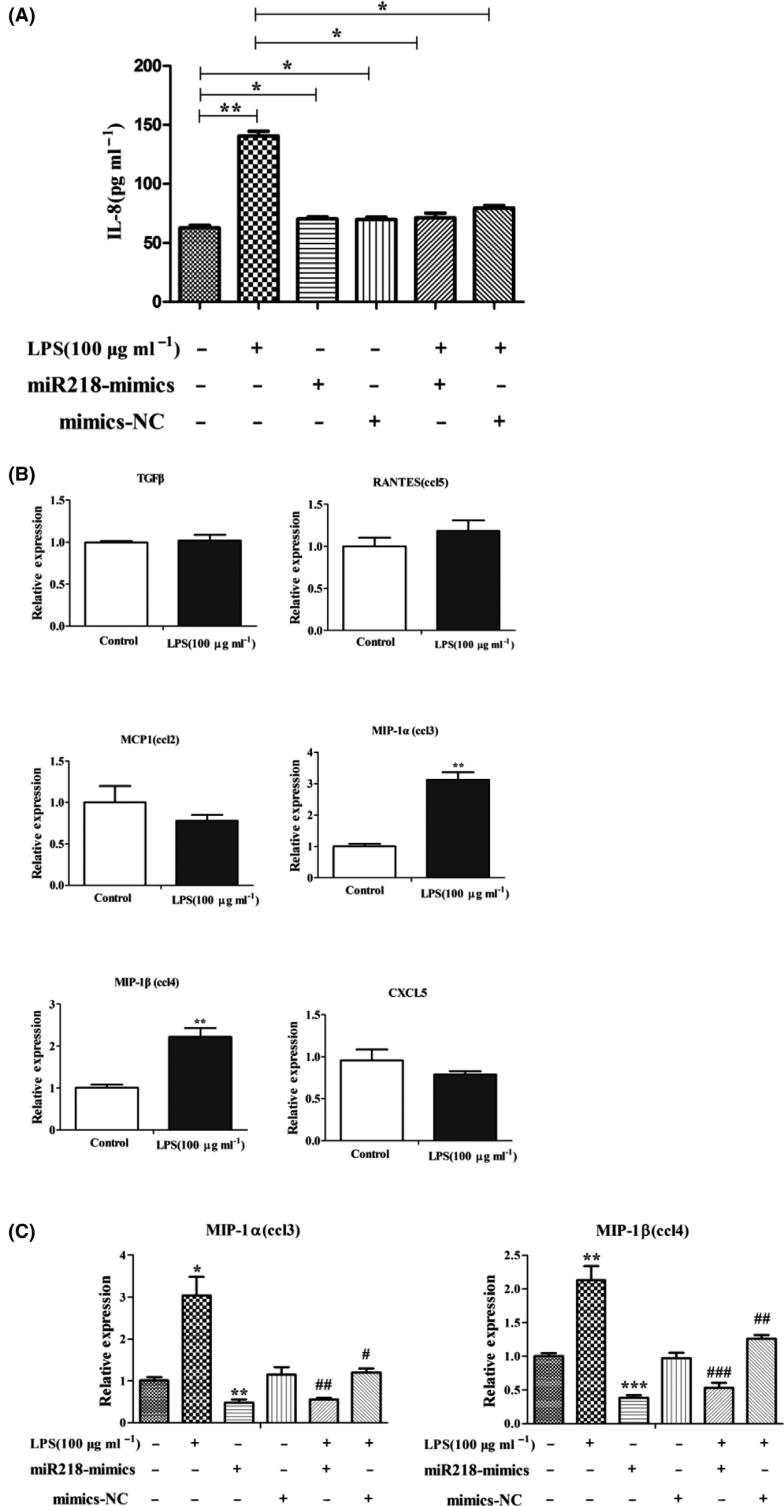
The effect of miR‐218 on the release of chemokines from endometrial epithelial cells. A. The effect of miR218 mimics or NC transfection on the release of IL‐8 induced by LPS administration. Data are represented with mean ± standard deviation from three independent experiments, * P < 0.05, **P < 0.01. B. mRNA expression of chemokines *TGF‐β*, *RANTES*, *MCP1*, *MIP‐1α*, *MIP‐1β* and *CXCL‐5* in LPS‐treated endometrial epithelial cells after 24 h as detected with qRT‐PCR. Data are represented with mean ± standard deviation from three independent experiments, **P < 0.01. C. The effect of miR‐218 mimics or NC transfection on the expression of chemokines *MIP‐1α* and *MIP‐1β* induced by LPS administration. Data are represented with mean ± standard deviation from three independent experiments, *P < 0.05 vs. Control group; **P < 0.01 vs. Control group; ***P < 0.001 vs. Control group; ^#^ *P* < 0.05 vs. LPS group; ^##^ *P* < 0.01 vs. LPS group; *^###^P* < 0.001 vs. LPS group.

miR‐218 mimic transfection had no significant effect on IL‐8 secretion but significantly reduced LPS‐induced IL‐6, TNF‐α and IL‐1β secretion (Fig. 5A, *P* < 0.05). In addition, we also screened the expression of other chemokines. There was no significant change in the mRNA expression of chemokines *TGF‐β*, *RANTES*, *MCP‐1* and *CXCL‐5* in the LPS‐induced endometrial epithelial inflammatory model. However, the expression of *MIP‐1α* and *MIP‐1β* was significantly increased (Fig. 5B, *P* < 0.01). The transfection of miR‐218 mimics reduced the expression of LPS‐induced chemokines *MIP‐1α* and *MIP‐1β* (Fig. 5C, *P* < 0.01).

## Discussion

It is well known that it takes 20–30 days for the uterus to recover and the constitution to recover after the calves are born. Most dairy cows show their first noticeable oestrus in 30–70 days after parturition. In this experiment, the cows with no oestrus and rectal examination with corpus luteum prominently protruding from the surface of the ovary within 21–30 days after delivery were selected as the research subjects. This study showed by *in situ* hybridization that the inflammation of the endometrium affected the localization of miR‐218 in the uterine tissue, restricting its expression to uterine gland tissue. Meanwhile, the qRT‐PCR results indicated that endometritis also affected the amount of the miR‐218 expression in uterine tissue, which is consistent with our previous high‐throughput sequencing results. Coincidentally, the downregulation of miR‐218 contributed to epithelial‐mesenchymal transition and tumour metastasis in lung cancer (Shi, *et al.*, 2017) and the proliferation of non‐small‐cell lung carcinoma cells (Yang, *et al.*, 2019). However, the uterus is a special organ that is regulated by ovarian steroid hormones during different estrous cycles in mammals. Related studies have also found that ovarian hormones regulate the function of miRNAs and immune system in the uterus (Edey, *et al.*, 2018; Li, *et al.*, 2020). Therefore, we developed a model *in vitro* for endometritis with LPS and chose the healthy cows and cows with endometritis in a consistent ovarian state as the research subjects to eliminate the effect of the stage of the oestrus cycle on miR‐218 and the release of cytokines.

Lipopolysaccharide, a component of the outer membrane of Gram‐negative bacteria cell walls, has been widely used in the study of inflammatory models for acute lung injury, mastitis, endometritis and so on (Li, *et al.*, 2015; Wu, *et al.*, 2016). This revealed that the secretion of pro‐inflammatory cytokines such as IL‐1β and TNF‐α increases significantly during LPS stimulation (Wu, *et al.*, 2017). To analyse this further, we developed a model *in vitro* for endometritis with LPS for displaying an increase in referential immune factors. The expression of miR‐218 was also reduced in LPS‐treated endometrial epithelial cells, which is consistent with the results *in vivo*.

miRNAs have attracted notable attention in many research fields since they were first discovered in exosomes. Here, we successfully obtained exosomes from the uterine fluid of healthy cattle and those with endometritis, and confirmed their morphology by electron microscopy. The expression of miR‐218 in the exosomes obtained from inflammatory cows was significantly lower than that from the exosomes derived from the uterine cavity fluid of healthy cows, which is consistent with the findings from tissues and LPS‐treated endometrial epithelial cells. Because the uterine cavity fluid exosomes from cows with endometritis can be taken up by endometrial epithelial cells and LPS‐treated endometrial epithelial cells, they can be used to transport intracellular miRNAs. Related research reported that miR‐128 can be delivered via exosomes to increase the chemosensitivity of oxaliplatin‐resistant colorectal cancer (Liu, *et al.*, 2019).

It was previously reported that the NF‐κB pathway can activate the transcription of pro‐inflammatory cytokines (Wu, *et al.*, 2017). Related study confirmed that miR‐218 suppressed the progression of cervical cancer via the LYN/NF‐κB signalling pathway (Xu, *et al.*, 2018). In addition, miR‐218 plays an important role in regulating the osteoclast differentiation and inflammation response in rats with periodontitis (Guo, *et al.*, 2019) and the development of human natural killer cells (Victor, *et al.*, 2018). Here, miR‐218 suppressed the activation of NF‐κB and the LPS‐induced secretion of TNF‐α, IL‐1β and IL‐6. However, there was no significant effect on IL‐8 secretion. IL‐8 is an important neutrophil‐specific chemokine (Hotamisligil, 2006), but IL‐6, TNF‐α and IL‐1β are major pro‐inflammatory factors. Therefore, our results are consistent with those of published literature.

Endometrial epithelial cells can recruit immune cells to respond to inflammation by releasing chemokines. To explore which chemokines are affected by miR‐218, we detected the mRNA expression of *TGF‐β*, *RANTES*, *MCP‐1*, *CXCL‐5*, *MIP‐1α* and *MIP‐1β* under LPS administration. LPS induced the expression of *MIP‐1α* and *MIP‐1β*, while miR‐218 could inhibit this activation to some extent. Hence, we infer that the high expression of miR‐218 can inhibit the activation of Mip‐1 in the healthy bovine uterus. In case of endometritis, the decrease of miR‐218 also reduces the inhibition of Mip‐1, enhances the chemotaxis of immune cells, and is conducive to the elimination of pathogenic bacteria and the maintenance of uterine health. Related studies also confirmed that MIP‐1 protein is inducible in the most immune cells in response to various pro‐inflammatory stimuli, and also a potent chemoattractant for innate and adaptive immunity (Menten, *et al.*, 2002; Maurer and von Stebut, 2004). MIP‐1 activates the chemokine receptors CCR‐1 and CCR‐5, which initiate diverse cellular responses that regulate both acute and chronic inflammation. Moreover, the MIP‐1α knockout mice showed altered inflammatory reactions in response to infection by various viral and bacterial pathogens (Cook, *et al.*, 1995; Sato, *et al.*, 1999; Lindell, *et al.*, 2001). Therefore, MIP‐1 plays a very important immunomodulatory role in cows with endometrial inflammation.

**Fig. 6:**
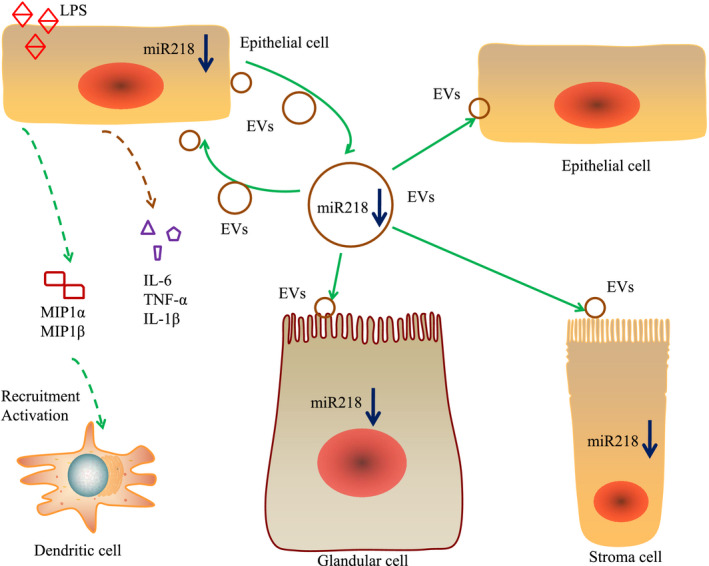
The updated model for indicating how exosome‐derived uterine miR‐218 isolated from cows with endometritis regulates the release of cytokines and chemokines.

In summary, the release of immune factors and chemokines in EECs and the recruitment of inflammatory cells, on one hand, are enhanced by inhibiting miR‐218 in case of endometrial inflammation; on the other hand, the amount of miR‐218 within the exosomes released by EECs is also reduced, corresponding to the decreased uptake by adjacent healthy cells, inflammatory cells and stromal cells (Fig. 6). Our findings extend the information about how EECs affect inflammation and provide a potential biomarker for the detection of endometritis.

## Experimental procedures

### Bovine uterus collection and exosome isolation

This study was performed in accordance with the guidelines of the Animal Ethic Committee of Beijing University of Agriculture. Bovine uteri were obtained from the slaughterhouse. Before slaughtered, the postpartum holsterin cows from Beijing Shun Sunshine Farm were monitored by rectal temperature measurement and rectal examination for uterine rejuvenation on Days 1, 7, 14, 21 and 30 after delivery, combined with the daily diseases and medication conditions of dairy cows. The bovine (parity 2–4, body condition score 3.25–4.0) uterus without other diseases such as mastitis, hoof disease, dermatitis and postpartum paralysis (Archbold, *et al.*, 2012), whose body temperature was lower than 39.5°C and the mucopurulent or purulent secretions secreted through vagina reaching the level 3 of vaginal mucus secretion in bovine endometritis were collected at 21–30 days postpartum as the clinical endometritis group (Williams, *et al.*, 2005). In addition, those healthy bovine uteruses without disease (vaginal endoscopy without mucopurulent discharge) but with normal body temperature, 21–30 days postpartum served as the control group. The cows both in healthy and in endometritis group have no oestrus, and corpus luteum prominently protruding from the surface of the ovary within 21–30 days after delivery with rectal examination. After being sent to the laboratory, five uteruses from healthy cows and five from ones with endometritis were dissected so as to observe the inflammatory state and then their secreted mucus was gently scraped from the uterine wall for Diff rapid cell staining (Solarbio, G1540, Beijing, China) to count the proportion of PMN (Polymorphonuclear) in the uterine cavity fluid. After the correctness of the uterine samples from healthy bovine uteruses and the cows with endometritis were determined, the cavities were rinsed with 10 ml exosome‐free PBS for 3 times. The PBS used for rinsing the cavities was then centrifuged at 3000 *g* and at 4°C for 15 min, and the supernatant was aspirated and stored at −80°C.

Exosomes were isolated from the uterine cavity fluid according to the manufacturer’s recommendations of the Exosome Extraction Kit‐Body Fluid (Bebo Bio, Shanghai, China). The uterine cavity fluid samples were thawed and placed on ice at 4°C; then, 10 ml of the sample was transferred to a new 15 ml conical tube. The sample was centrifuged at 3000 *g* and at 4^o^C for 15 min. The precipitation was discarded and the supernatant collected and then carefully transferred into another clean centrifuge tube. The subsequent samples were centrifuged at 10 000 *g* and at 4°C for 20 min. Next, the precipitation was discarded and the supernatant collected and then carefully transferred into another clean centrifuge tube. 1 ml extract A was added to 4 ml supernatant, the lid of the centrifuge tube was closed before and the liquid was mixed upside down for about 1 min. After the mix was kept in the refrigerator at 2–8^o^C overnight, it was centrifuged at 10 000 *g* and at 4°C for 60 min. After the supernatant was carefully removed and the precipitate collected, 50 μl of the preserved fluid with exosomes was precipitated and then resuspended to obtain the uterine cavity fluid exosomes.

### Morphology of exosomes by electron microscopy

The sample of 10 μl was added onto the copper grid to precipitate for 1 min, and the floating liquid was absorbed with filter paper. Then, 10 μl of uranyl acetate (phosphotungstic acid) was added onto the copper grid to precipitate for 1 min, and the floating liquid was absorbed with filter paper. After it was dried for 10 min at room temperature, and electron microscopy imaging was performed at 80 kV.

### PKH26 staining for exosomes

The PKH26 Red Fluorescent Cell Linker Kits (Sigma, Santa Clara, CA) were used for lipid bilayer labelling. Exosomes were first resuspended in 100 μl Diluent C. A dye solution (4 × 10^6^ M) was prepared by adding 0.4 μl PKH26 ethanolic dye solution to 100 μl Diluent C. 100 μl of the exosome suspension was then mixed with 100 μl of the dye solution by pipetting. After the cell incubation and dye suspension with periodic mixing operations for 1–5 min, the staining was stopped by adding 200 μl serum and incubation for 1 min. The stained exosomes were finally washed twice with 1 × PBS, and suspended with 10 μl PBS in a fresh sterile conical polypropylene tube. After that, the confocal microscopy was used to detect the internalization of the fluorescently labelled exosomes in EECs after 12 h incubation.

### Cultivation, identification and lipopolysaccharide (LPS) challenge of bovine endometrial epithelium cell lines (EECs‐BEND)

EECs (BEND cell line purchased from ATCC Cell Bank, Beijing, China) were cultured in DMEM/F12 (Invitrogen Inc., Carlsbad, CA, USA) at 1 × 10^5^ cells per well, and supplemented with 10 % FCS (Gibco , USA) streptomycin in a 5% CO_2_ atmosphere at 37°C. Immunofluorescent detection was used for the purity identification of endometrial epithelial cells. EECs at Passage 3 were cultured in 24‐well plates for 24 h. After the immunofluorescent staining of keratin, EECs were fixed in 4% paraformaldehyde for 30 min and then permeabilized with 0.1% Triton X‐100 in PBS for 15 min, subsequently blocked for 1 h with 5% BSA in PBS at room temperature, and co‐incubated with anti‐keratin antibody (Abcam, 1:500 dilution, ab111599) at 37°C for 2 h, respectively. After being washed and then incubated with anti‐rabbit secondary antibody (Invitrogen, A21206; 1:500 dilution) at 37°C for 1 h, the nuclei were stained with 4′, 6‐diamidino‐2‐phenylindole (DAPI) for 3–5 min. The fluorescent signals were examined with the fluorescence microscope (Olympus, Tokyo, Japan). The EECs which were identified to be more than 90% pure were challenged with the different doses (0–100 µg ml^−1^) of LPS (*E. coli* 0111: B4; Invitrogen) for different periods of time (0–48 h). After that, the cells were treated with 10 μl CCK‐8 and then incubated for additional 2 h. The absorbance (OD) of each hole was measured at 450 nm with a micrometer.

### Cell transfection

EECs or LPS‐treated EECs were seeded into 6‐well plates and transfected by use of Lipofectamine 2000 (Invitrogen) and Opti‐MEM (Gibco), according to the manufacturers’ instructions. For miRNA upregulation and downregulation, a 100‐pmol dose of miR‐218 mimics and NC was used. In addition, EECs were harvested after 24 h after transfection to isolate total RNA or total cell lysate or assess the transfection efficiency. The miR‐218 mimics and NC sequences were designed and made by Shanghai Gemar Co., LTD as follows: 5’‐UUGUGCUUGAUCUAACCAUGUG‐3’(miR‐218 mimics, sense), 5’‐CAUGGUUAGAUCAAGCACAAUU‐3’(miR‐218 mimics, anti‐sense), 5’‐UUCUCCGAACGUGUCACGUTT‐3’ (NC, sense), and 5’‐ACGUGACACGUUCGGAGAATT‐3’ (NC, anti‐sense).

### In situ hybridization

The uterine tissue was taken out from the −80°C freezer, thawed at room temperature and stored in 4% paraformaldehyde for fixation, before the frozen sections were prepared. The frozen section samples were treated with a mixture of 30% H_2_O_2_ and methanol (1:9) at room temperature for 10 min. After being washed with DEPC for 3 times, about 1 min each time, the sections were placed in a wet box made of 5 × SSC (pH: 7.5) (35 ml) mixed with formamide (35 ml), dropped onto the tissue with 0.25% hydrochloric acid before standing at room temperature for 15 min, and washed with DEPC twice, 1 min each time. The proteinase K was covered with tissue, hybridized in a molecular hybridization instrument at 37 ^o^C for 1 h, and the protein K was stopped by washing with a 0.2% glycine wash solution for 1 min. The sample was washed with PBS twice, for 1 min each time, and then with 5 x SSC (pH: 7.5) twice, 1 min each time. The sections were placed in a wet box, the tissues were covered with the pre‐hybridization solution, and they were pre‐hybridized at 65^o^C for 1 h. The sections were covered with a 500 ng ml^−1^ probe and reacted in the dark in the hybridization apparatus at 62–70°C for 48 h. After they were washed with 2 x SSC (pH: 7.5) once, for 1 min each time, the formamide was mixed with 4 × SSC (pH: 4.5) 1:1, and the mixture was washed at 60°C for three times, 20 min each time. They were washed with PBS at room temperature five times, for 1 min each time. The sections were placed in a wet box, and the blocking solution was covered with the blocking solution and reacted at room temperature for at least 30 min. The anti‐digoxigenin antibody of biotinylated mice was added dropwise at 37°C for 2 h, and PBS was washed for three times, 5 min each time. FITC‐labelled antibody was added dropwise, and the reaction was incubated in the dark at 37°C for 1 h, and washed with PBS 3 for times, 5 min each time. DAPI was stained with nuclei and washed with PBS for three times, 5 min each time. Finally, the anti‐quenching agent was added dropwise to a cover slip, a nail polish seal and a laser confocal microscope. The miR‐218 gene probe was designed and synthesized by Shanghai Gemar Pharmaceutical Technology Co., LTD as follows: 5’‐CACATGGTTA GATCAAGCACAA‐3’(miR‐218) and 5’‐GTGTAACACGTCTATACGCCCA‐3’(NC).

### Real‐time detection of RT‐PCR (qRT‐PCR)

Total RNA of the exosome and EECs of uterine tissues were extracted with the Trizol (Invitrogen Inc.), and cDNA was synthesized with the Prime Script RT Reagent Kit (TaKaRa Bio Inc., Dalian, China), according to the manufacturers’ protocols. (i) miR‐218 expression in healthy bovine uteruses or those with endometritis, the miR‐218 and GAPDH gene primers were designed and made by Shanghai Gemar Pharmaceutical Technology Co., LTD as follows: 5’‐GCCGCTTGTGCTTGATCTA‐3’ (miR‐218, sence), 5’‐AGAGCAGGGTCCGAGGAT‐3’(miR‐218, anti‐sence), 5’‐GGCGTGAACCACGAGAAGTA‐3’(GADPH, sence) and 5’‐GGCGTGGACAGTGGTCATAA‐3’(GADPH, anti‐sence). (ii) miR‐218 expression in EECs or LPS‐treated EECs. (iii) miR‐218 expression in healthy bovine uterus cavity derived exosomes or endometritis bovine uterus cavity derived exosomes. (iv) miR‐218 expression in EECs or miR‐218 mimics transfection EECs. (v) *TGF‐β, RANTES, MCP‐1, CXCL‐5, MIP‐1α* and *MIP‐1β* mRNA expressions in EECs or LPS‐treated or miR‐218 mimics or NC transfection EECs. The procedures were operated as described previously (Wang, *et al.*, 2019). The GenBank accession numbers and primer sequences of *TGF‐β, RANTES, MCP‐1, CXCL‐5, MIP‐1α* and *MIP‐1β* and *β‐actin* were summarized in Table 1.

**Table 1:** The Primer Sequences Used for qRT‐PCR.

Sequence(5′–3′)	Gene Name	Size (bp)
cacgtggagctgtaccagaa	TGF‐β NM_001166068.1	101
acgtcaaaggacagccactc		
cacccacgtccaggagtatt	RANTES (ccl5) NM_175827.2	110
cccacttcttctctgggttg		
cagaagagtcaccagcagca	MCP‐1 (ccl2) NM_174006.2	106
ggagtcctggacccatttct		
agcctggtgtcatcttccag	MIP‐1α (CCL3) NM_174511.2	93
gctccaggtcggtgatgtat		
tgactgtcctgtccctcctc	MIP‐1β(ccl4) NM_001075147.2	120
gaggaatcttccgcagagtg		
cccaaaacggtcagtgatct	CXCL‐5 NM_174300.2	100
ccagacagacttcccttcca		
ATCCTGCGGCATTCACGAA	ACTB NM_173979.3	154
TGCCAGGGCAGTGATCTCTT		

### Western blot detection

(i) Detection of exosome markers by Western blot. The exosomes obtained from the uterine cavity fluid were detected by Western blot for the purpose of the expression of CD9 (Anti‐CD9 antibody, Abcam, 1:1000 dilution, ab19761) protein in the exosomes. (ii) Detection of p65 (Anti‐NF‐kB p65 Monoclonal Antibody, Solarbio, 1:500, K200045M) and phosphorylation‐p65 (Anti‐NF‐kB p65 (Phospho S536), Abcam, 1:1000, ab86299) protein expression were operated as described previously (Wang, *et al.*, 2019).

### Phosphorylated p65 immunofluorescent staining

Endometrial epithelial cell, LPS‐treated EEC and LPS‐treated EEC with miR‐218 transfection were fixed in 4% paraformaldehyde for 30 min, and then permeabilized with 0.1% Triton X‐100 in PBS for 15 min, subsequently blocked with 5% BSA in PBS at room temperature for 1 h, and co‐incubated with anti‐ NF‐kB p65 (phospho S536) (Abcam, 1:1000, ab86299) at 37ºC for 2 h. After washing, it was incubated with secondary antibody at 37°C for 1 h, and the nuclei were stained with 4′, 6‐diamidino‐2‐phenylindole (DAPI) for 3–5 min. The fluorescent signals were examined under a fluorescence microscope (Olympus).

### ELISA detection

The concentrations of IL‐6, IL‐8, TNF‐α and IL‐1β in the culture media of LPS‐treated or miR‐218 mimics or NC transfection EECs were assayed with the ELISA kits (BD Bioscience, San Jose, CA, USA), according to the manufacturer's recommendations. The optical densities at 450 nm of each well were determined by use of a micro‐plate reader (Model 680, Bio‐Rad, Hercules, CA, USA).

### Statistical analysis

The experimental results were derived from at least three independent experiments and presented as Mean ± Standard Deviation (*M* ± SD). The data were analysed with one‐way ANOVA, followed by Fisher’s least significant different test (Fisher LSD) and independent‐samples *t* test with the SPSS (Statistical Package for the Social Sciences) software (version 16.0; SPSS, Inc., Chicago, IL, USA). The differences were considered to be significant when *P* < 0.05.

## Conflict of interest

None of the authors has any conflict of interest in publishing this study.

## Authors’ contributions

XGW and TTX performed the experiments, collected and interpreted the data. XHS, BFF and XLQ provided initial help with analysis. KX, FL and JD collected the samples. XGW and YG conceptualized and wrote the manuscript. HMN reviewed the manuscript prior to publication.
